# “Hot-spotting” to improve vaccine allocation by harnessing digital contact tracing technology: An application of percolation theory

**DOI:** 10.1371/journal.pone.0256889

**Published:** 2021-09-22

**Authors:** Mark D. Penney, Yigit Yargic, Lee Smolin, Edward W. Thommes, Madhur Anand, Chris T. Bauch

**Affiliations:** 1 Perimeter Institute for Theoretical Physics, Waterloo, Ontario, Canada; 2 Department of Applied Mathematics, University of Waterloo, Waterloo, Ontario, Canada; 3 Department of Physics and Astronomy, University of Waterloo, Waterloo, Ontario, Canada; 4 Vaccine Epidemiology and Modeling, Sanofi Pasteur, Toronto, Ontario, Canada; 5 Department of Mathematics and Statistics, University of Guelph, Guelph, Ontario, Canada; 6 Department of Mathematics and Statistics, York University, Toronto, Ontario, Canada; 7 School of Environmental Sciences, University of Guelph, Guelph, Ontario, Canada; Texas A&M University College Station, UNITED STATES

## Abstract

Vaccinating individuals with more exposure to others can be disproportionately effective, in theory, but identifying these individuals is difficult and has long prevented implementation of such strategies. Here, we propose how the technology underlying digital contact tracing could be harnessed to boost vaccine coverage among these individuals. In order to assess the impact of this “hot-spotting” proposal we model the spread of disease using percolation theory, a collection of analytical techniques from statistical physics. Furthermore, we introduce a novel measure which we call the efficiency, defined as the percentage decrease in the reproduction number per percentage of the population vaccinated. We find that optimal implementations of the proposal can achieve herd immunity with as little as half as many vaccine doses as a non-targeted strategy, and is attractive even for relatively low rates of app usage.

## Introduction

Vaccine allocation decisions for pandemic infectious diseases such as COVID-19 must weigh a complex set of competing factors based on transmission characteristics [1], differences in vulnerability across demographic groups [2, 3], and the principle of reciprocity, which states that those who accept the greatest risks to mitigate the effects of the pandemic should be vaccinated first [4]. The initially limited supply of vaccines that is expected during a pandemic highlights the importance of these trade-offs, necessitating careful decisions about their use [5].

One particular trade-off that has been explored in the influenza modelling literature is whether to use the vaccine to protect vulnerable groups, or to reduce community transmission [6]. Recent models of SARS-CoV-2 transmission dynamics suggest that in certain parameter regimes and for COVID-19 vaccines that have some effectiveness against both disease and infection, vaccinating to block transmission may prevent more deaths than targeting vulnerable age groups [1, 7, 8]. The strong potential effects of using vaccines to interrupt transmission, and the initially restricted supply of vaccines that is expected during a pandemic, motivate us to ask the following question: How can one best allocate a limited supply of vaccines in order to achieve the greatest reduction in disease transmission?

Allocation of vaccines is often based on age or other demographic factors, since we know that contact patterns differ between demographic groups. However, heterogeneity within demographic groups is much greater than the differences between them [9]. Empirical evidence from the ongoing COVID-19 pandemic has pointed to significant overdispersion in the number of secondary cases [10] which is not entirely explained by differing viral loads [11, 12]. Because individuals with more contacts (and, thus, exposure) have the potential to cause more transmission than individuals with low exposure to others, targeting these individuals for vaccination has been found to be highly effective in mathematical models [13–15]. But such strategies are difficult to implement in practice since public health authorities only have information on broad demographic or regional factors upon which to act.

It is exactly in this regard that digital contact tracing technologies have opened up a new avenue for public health interventions. The core functionality of Bluetooth-based exposure notification apps is the creation of an encounter log between app users. This encounter log is useful for more than just exposure notification: It also quantifies the user’s exposure to others. Said another way, these apps are also sensors that measure the duration of exposure–an epidemiologically significant quantity.

We propose that Bluetooth exposure notification apps can be harnessed to improve vaccine program effectiveness. These apps could be used to increase vaccine coverage among individuals with very high exposure rates, and thereby provide a highly efficient means to limit transmission in the population. We present mathematical modelling results in support of our proposal based on percolation theory: a collection of analytical techniques from statistical physics [16] that have been applied to material sciences [17], the spread of forest fires [18, 19], infectious diseases [20] and other areas. We assume a public health goal of interrupting transmission as efficiently as possible, and where vaccines can prevent transmission. The tools we developed show that the improved efficiency gained by piggy-backing vaccine allocation strategies on top of exposure notification apps is a robust phenomenon, as it derives its power from the very heterogeneities in contact patterns that shape the spread of infectious diseases.

### Bluetooth exposure notification

In March 2020, COVID Watch released a white paper detailing an anonymous Bluetooth-based system that exploits the ubiquity of Android and iOS smartphones to support contact tracing [21]. The idea has seen widespread adoption, with nearly every developed country having incorporated it into their digital contact tracing solutions. Both the Google/Apple and BlueTrace frameworks are also based on this technology, the former of which is available throughout North America and the European Union and the latter in Singapore and Australia. Uptake has varied significantly between countries (Table 1), with most countries having made the usage of the app a personal choice. Singapore is a notable exception to this, having achieved high uptake rates due to mandatory use of the software.

**Table 1:** Exposure notification app download rates in selected countries based on official sources.

Country	Downloads per 100 people
Canada [22]	23
Germany [23]	36
Italy [24]	19
New Zealand [25]	60
Singapore [26]	>90

Protecting users’ privacy was a fundamental principle underlying the design process. Every 10-20 minutes, anonymous tokens are exchanged between app users who are in close proximity to one another. Each user’s device stores these tokens in an encounter log. The data stored by the app is able to determine neither the number of contacts nor the duration of any individual contact. Instead, the number of tokens logged over a given time frame is a measure of the user’s *total exposure time* to others: the sum, over each contact, of the duration of that contact.

Theoretical vaccination strategies that vaccinate individuals in accordance to their number of contacts have previously been studied in the context of immunizing scale-free networks [13]. Using exposure notification apps one can implement strategies which preferentially vaccinate individuals in accordance with their total exposure time. In what follows we will develop a model for more general vaccination strategies which depend on both the number and duration of contacts. We present this more general model not just because it is no more difficult, but also because such strategies could, with changes to the exposure notification systems, be implemented without the centralized collection of user data. Of course, any changes to the protocols come with concerns around privacy and data protection, and so for our simulations we restrict ourselves to considering a hot-spotting strategy that can be implemented without introducing any new privacy concerns.

The means by which the decentralized encounter log is used to facilitate contact tracing is by the central collection and communication of those tokens associated with individuals who have tested positive for COVID-19. In the Google/Apple framework public health authorities provide COVID-19 positive individuals with a code which, when voluntarily entered in the app, uploads their tokens to a central repository.

This same system can be used for ring vaccination strategies, which have been used in campaigns against smallpox [27] and Ebola [28]. However, there would have to be an important change in the type of information sent to the central repository from infected users. Instead of sending their own tokens, they would share the tokens they’ve collected from other users. With the same change, neighbor vaccination strategies [29] are also possible. Despite the fact that ring vaccination has already been studied using percolation theory [30, 31], and neighbor vaccination strategies certainly can in principle be, we restricted our attention to exposure-based strategies for the same reasons as in the preceding paragraph.

### Weighted percolation theory

Many infectious diseases spread through close contact, and contact patterns in human populations display a high degree of heterogeneity [32, 33]. One successful approach to understanding the impact of heterogeneity is to model the spread of infectious disease as a percolation process on the network of contacts [34–36]. In a series of foundational works [37, 38], Newman developed analytical techniques based on *probability generating functions*. In this formalism, the information needed from the network is the degree distribution {*p*_*k*_}, where *p*_*k*_ is the fraction of the individuals in the network which have *k* contacts or, equivalently, have degree *k*. Using the probability generating function of the degree distribution, *G*_0_(*x*) = ∑_*k*_ *p*_*k*_ *x*^*k*^, a standard technique for studying discrete probability distributions [39], Newman derived formulas for key epidemiological quantities in terms of *G*_0_ and its derivatives.

More concretely, the infectious process is modelled as an instance of bond percolation. The disease spreads through occupied edges, and in Newman’s work each edge has a uniform probability *T* of being occupied. In the language of percolation theory, the basic reproduction number *R*_0_ is the expected number of occupied edges attached to an infected vertex, with one edge removed from each infected vertex to account for the individual which infected the vertex in question. It was shown in [38] that this expectation value can be computed in terms of *G*_0_ as

$$\begin{array}{c} R_{0}=T\frac{G_{0}^{\prime \prime }\left(1\right)}{G_{0}^{\prime }\left(1\right)}=T[\mu (1+\frac{\sigma^{2}}{\mu^{2}})-1] \end{array}$$

where *μ* and *σ* are, respectively, the mean and standard deviation of the degree distribution. Let us briefly summarize the derivation for those readers who may be unfamiliar with these techniques.

First, the probability that a vertex of degree *k* has *n* ≤ *k* occupied edges attached to it is (kn)(1-T)k-nTk. This fact allows one to determine that the distribution of occupied degree in the network has probability generating function *G*_0_(*x*;*T*) = *G*_0_(1 − *T* + *Tx*). The basic reproduction number is the expectation value of a closely related distribution. Namely, the expectation must be taken over only the infected vertices in the network. In Newman’s work it is assumed that the network of contacts is a random graph, an assumption implying that the desired distribution has probability generating function $G_{1}\left(x;T\right)=\frac{1}{\mu }G_{0}^{\prime }\left(1-T+Tx\right)$. A general property of probability generating functions is that the expected value of a distribution is simply the derivative of its generating function evaluated at *x* = 1. So, the basic reproduction number is calculated as $R_{0}=G_{1}^{\prime }\left(x;T\right)$, leading to the formula stated above.

In order to model vaccine allocation strategies based on exposure notification apps it is necessary to incorporate contact duration into the percolation model. To do this we consider the contact network to be weighted: each edge is further equipped with a weight *w*. The transmission probability *T* along an edge for the percolation process is assumed to depend on that edge’s weight, so that the single parameter is replaced with a distinct *T*_*w*_ for each weight *w*.

For our purposes in this paper the weight represents the number of time-steps over which the contact took place. If one assumes that in each time-step there is an independent probability *T*_1_ of transmission, the transmission probability after *w* time-steps is *T*_*w*_ = 1−(1 − *T*_1_)^*w*^.

However, the weights could represent any factor which influences the transmission probability along an edge, such as the nature of the contact, the setting, or the presence or absence of PPE. Unless stated otherwise, our analytical results hold for this general interpretation of weights.

In a weighted network we represent the configuration of edges around each vertex by a *generalized degree*, denoted ***k***. This is a vector having an entry for each of the possible weights appearing in the network. For a vertex of generalized degree ***k***, the entry corresponding to weight *w*, denoted *k*_*w*_, is the number of contacts having weight *w*.

To generalize Newman’s results to the weighted setting we introduce a *multivariable generating function* for the distribution of generalized degrees. Since we are keeping track of the number of edges of each weight separately, the generating function needs a variable *y*_*w*_ for each weight *w* appearing in the network. Let *q*_***k***_ denote the fraction of vertices in the network having generalized degree ***k*** and let ***y*** denote the vector of variables *y*_*w*_. Then the multivariable generating function of the network is

$$\begin{array}{c} Q\left(y\right)=\sum_{k}q_{k}y^{k}\;,\;y^{k}=\prod_{w}y_{w}^{k_{w}}\;. \end{array}$$

We derive the basic reproduction number using the exact same approach as Newman, except starting from the multivariable generating function. Specifically, the distribution of occupied degree has probability generating function,

$$\begin{array}{c} G_{0}\left(x;T\right)=Q\left(1-T+Tx\right)\;, \end{array}$$

where ***T*** is the vector composed of the transmission probabilities *T*_*w*_. Furthermore, we introduce

$$\begin{array}{c} G_{1}\left(x;T\right)=\frac{G_{0}^{\prime }\left(x;T\right)}{G_{0}^{\prime }\left(1;T\right)} \end{array}$$

as the probability generating function for the number of other occupied edges attached to a vertex reached by following a random occupied edge (cf. [38]). By definition, the basic reproduction number *R*_0_ is precisely the mean of this final distribution, i.e. $R_{0}=G_{1}^{\prime }\left(1;T\right)$.

One can express *R*_0_ directly in terms of the generating function *Q*(***y***). This is accomplished by introducing the differential operator ∇_***T***_, defined as

$$\begin{array}{c} \nabla_{T}Q\left(y\right)=\sum_{w}T_{w}\;\frac{\partial Q(y)}{\partial y_{w}}\;. \end{array}$$

Then, we find

$$\begin{array}{c} R_{0}=\frac{\nabla_{T}^{2}Q\left(1\right)}{\nabla_{T}Q\left(1\right)}\;. \end{array}$$

Alternatively, this result can be rewritten in a form which is more amenable to computation,

$$\begin{array}{c} R_{0}=\frac{\mathrm{Av}[{\left(\sum_{i}T_{w(i)}\right)}^{2}]-\mathrm{Av}[\sum_{i}T_{w(i)}^{2}]}{\mathrm{Av}[\sum_{i}T_{w(i)}]}\;. \end{array}$$

In this equation, the averages Av[⋯] are taken over all vertices in the network and the sums ∑_*i*_ are taken over all contacts *i* of a vertex, with *w*(*i*) being the weight of that contact. Note that this formula holds regardless of the interpretation of the weights as contact duration.

### Modelling vaccination

We model vaccination as a stochastic process wherein a vertex of generalized degree ***k*** has a probability *v*(***k***) of being vaccinated. Vaccination modifies the original contact network by *removing vaccinated vertices*, as we assume that a vaccinated individual can neither become infected nor infect others. The model can readily incorporate imperfect efficacy and results in a simple rescaling of the main results shown below. Indeed, in the language of percolation theory vaccination is an instance of site percolation, and the combined spread of a disease and vaccination is modeled as a mix of bond and site percolation.

The formulas for the basic reproduction number *R*_0_ can be adapted to give the expected post-vaccination reproduction number. This adaptation requires two changes: Firstly, each vertex of generalized degree ***k*** has a probability 1 − *v*(***k***) of remaining in the network, that is, of being unvaccinated. Secondly, the changes to the generalized degree of the remaining unvaccinated individuals must be taken into account. Specifically, the probability that a weight *w* edge leads to an unvaccinated vertex is,

$$\begin{array}{c} \varphi_{w}=1-\frac{\mathrm{Av}[k_{w}v\left(k\right)]}{\mathrm{Av}[k_{w}]}\;. \end{array}$$

Hence, the expected post-vaccination reproduction rate is given by

$$\begin{array}{c} E[R^{v}]=\frac{\mathrm{Av}[(1-v(k))({\left(\sum_{i}{\tilde{T}}_{w(i)}\right)}^{2}-\sum_{i}{\tilde{T}}_{w(i)}^{2})]}{\mathrm{Av}[(1-v(k))\sum_{i}{\tilde{T}}_{w(i)}]}\;, \end{array}$$

where ${\tilde{T}}_{w}=T_{w}\phi_{w}$, and the averages Av[⋯] on the right-hand side run through all vertices in the network.

To compare two vaccine strategies in a context of limited supply we introduce a novel measure of the *efficiency* of a vaccination strategy:

$$\begin{array}{c} E^{v}=\frac{1-R^{v}/R_{0}}{V}\;, \end{array}$$

where *R*^*v*^ is the post-vaccination reproduction number and *V* is the fraction of the population that receives the vaccine. In other words, the efficiency of a strategy is the percentage decrease in the reproduction number per percentage of the population vaccinated.

In a stochastic process of vaccination, *V*, *R*^*v*^ and *E*^*v*^ are all random variables. In particular, the vaccine coverage rate *V* has the expected value E[V]=Av[v(k)] and the variance $\mathrm{Var}\left[V\right]=\frac{1}{N}\mathrm{Av}[v\left(k\right)(1-v(k))]$, where *N* is the size of the population. Therefore, for computing the expectation value of the efficiency *E*^*v*^ in a large population, we may neglect the variance in *V* and treat it as a fixed number at its mean value. Hence, we can write

$$\begin{array}{c} E[E^{v}]=\frac{1-E[R^{v}]/R_{0}}{E[V]}\;. \end{array}$$

One can compare any vaccination strategy to the uniform one, under which vaccines are distributed uniformly across the population to achieve a target vaccine coverage without taking into account any form of contact or exposure heterogeneity. For a uniform vaccination probability *v*, one would have $E[R^{v}]=(1-v)R_{0}$ and $E[E^{v}]=1$. Strategies for which the efficiency is less than 1 are regarded as inefficient, since the same number of vaccines could have achieved a greater impact if they were allocated uniformly. On the other hand, strategies with efficiency greater than 1 are promising candidates for a significant impact.

### The hot-spotting strategy

Here we propose a “hot-spotting” vaccination strategy, in reference to a fire-fighting practice that focuses on areas with intense fires [40]. This strategy aims to increase vaccine coverage among individuals with higher total exposure time, as determined by the Bluetooth apps. It operates without the central collection of any user data, as the selection of each user is decided locally by their device.

The hot-spotting strategy depends on a parameter *β* encoding the probability of success in a weighted coin-flip. Each user performs a virtual coin flip for each encounter stored in their encounter log over a fixed time period. The app selects those users who receive at least one success. Since the probability of obtaining at least one success on *n* weighted coin flips is 1−(1 − *β*)^*n*^, we see that users with a greater number of recorded encounters have a greater probability of being selected. Note that the extent to which high total exposure time individuals are preferentially selected can be increased by requiring a greater number of successes.

In order to estimate the impact and efficiency of our hot-spotting strategy on the whole population, we must take into account that not every person in the population uses the app in question. To this end, we assume that there is an app usage rate of *U* and that the app users are homogeneously distributed in the population. We also assume that there is no preferential attachment between app users. This implies that for a random individual with the contact structure ***k***, which is not necessarily registered in the app network, the probability for them being an app user and also being selected by the app is given by

$$\begin{array}{c} v\left(k;\beta ,U\right)=U(1-\prod_{w}\gamma_{w}^{k_{w}})\;, \end{array}$$

where

$$\begin{array}{c} \gamma_{w}=1-U+U{\left(1-\beta \right)}^{w}. \end{array}$$

We simulate an implementation of hot-spotting wherein individuals receive a vaccine if and only if they have been selected by the app, although the results are qualitatively similar if the app only increases the likelihood of vaccine acceptance among these individuals.

## Methods

### Model network

The model weighted contact network is based on contact data collected in the well-known POLYMOD study that surveyed over 7000 individuals across 8 European countries (Belgium, Germany, Finland, Great Britain, Italy, Luxembourg, the Netherlands, and Poland) between May 2005 and September 2006 [9]. Respondents kept a log of all contacts made on a single day noting, among other features, how long the contact lasted and how frequently that contact is made. This data captures normal contact patterns and doesn’t reflect any changes which can occur in response to an ongoing pandemic.

In the language of time-weighted networks, the inclusion of duration data means that the survey responses sample from the generalized degree distribution of the daily contact network. The network should capture the contacts made over the typical infectious period of the disease being modelled. For simplicity we choose a period of 14 days, as it aligns well with the frequency responses in the survey.

Using the daily contact data we must generate samples from the generalized degree distribution of the *fortnightly contact network.* We accomplish this by a bootstrapping technique. More precisely, for each contact recorded, respondents chose between 5 options concerning both the duration and the frequency of that contact as shown in Table 2. We can therefore represent each respondent’s contacts on that day in a 5 × 5 matrix whose (*i*, *j*) entry is the number of contacts recorded with frequency key *i* and duration key *j*. For respondent *n* we denote this matrix by *D*_*n*_.

**Table 2:** Possible responses on POLYMOD survey concerning frequency and duration of contact.

Keys	Frequency	Duration
(1)	daily	< 5 mins
(2)	1 − 2 times per week	5 − 15 mins
(3)	1 − 2 times per month	15 − 60 mins
(4)	less than once a month	1 − 4 hrs
(5)	first time	> 4 hrs

Our goal is to extrapolate fortnightly contact matrices *F*_*n*_ from the daily contact matrices *D*_*n*_. In our bootstrapping procedure, it is important that we distinguish between daily repeating contacts, which have frequency key (1), and *infrequent contacts*, which have frequency keys (2)-(5). We denote by *I*_*n*_ the matrix of infrequent contacts for respondent *n*. The matrix *I*_*n*_ is obtained from the same respondent’s *D*_*n*_ by setting the first row of daily repeating contacts to 0.

The matrices *F*_*n*_ are created by sampling from the daily contact matrices. Each sample is generated as follows:

1. Sample 14 respondents, *n*_1_, …, *n*_14_.
2. Set $F_{n}^{\prime }=D_{n_{1}}+I_{n_{2}}+\ldots +I_{n_{14}}$.
3. Produce *F*_*n*_ from $F_{n}^{\prime }$ by dividing each entry in the second row by 3 and rounding it to the nearest integer.

In words, we sample 14 daily contact logs from the survey. Then, we add together the 1-day duration-frequency matrices for each of the samples, excluding the daily repeating contacts from all but the first sample. Then, assuming that a frequency-key-(2) contact is seen 3 times in a fortnight, we divide the second row by three to account for repeated counting of the same contact.

Finally, we assign to each type of contact in the matrix *F*_*n*_ a certain weight. Each unit of weight is approximately 10 minutes of contact time, which corresponds to the token exchange rate of the exposure notification apps. We chose these weights to be given as in the following weight matrix:

$$\begin{array}{c} W=[\begin{array}{ccccc} 0 & 12 & 36 & 120 & 480\\ 0 & 3 & 9 & 30 & 120\\ 0 & 1 & 3 & 10 & 40\\ 0 & 1 & 3 & 10 & 40\\ 0 & 1 & 3 & 10 & 40 \end{array}] \end{array}$$

Hence, we obtain a list of generalized degrees sampled from a virtual weighted contact network, in which the set of possible weights is {1, 3, 9, 10, 12, 30, 36, 40, 120, 480}.

### Simulation

The simulations are based on 500 samples of fortnightly generalized degree distributions generated through the bootstrapping procedure described above. We interpret these 500 samples as an *observed cohort* of 500 individuals within a much larger population. As such, the recorded contacts are assumed to lie outside the observed cohort.

For these simulations we set the unit transmission probability at *T*_1_ = 0.000375 yielding a basic reproduction number (in the absence of vaccination) of *R*_0_ = 1.501. This value of *T*_1_ was chosen as the corresponding value of *R*_0_ is on the lower range of *R*_0_ estimates for the 2009 influenza A/H1N1 pandemic in the USA [41]. The expected efficiency is, in fact, quite insensitive to the value of *T*_1_, with the change of *τ* in *T*_1_ leading to a change in expected efficiency of $O(\tau^{2})$.

We select a set of parameters *β* for the vaccination probability function *v*(***k***;*β*, *U*) of the hot-spotting strategy (and a set of parameters *v* for the uniform strategy) to target a homogeneous distribution of the vaccinate rate *V* in the outcome over its range of possible values. 10,000 simulations are performed for each selected parameter. This accounts to a total of 990,000 simulations for the strategies with *U* = 100%. For the strategies with *U* = 20%, 40%, and 60%, we ran a total of 190,000, 390,000, and 590,000 simulations, respectively.

Each simulation consists of four steps:

1. The vaccination probabilities *v*(***k***;*β*, *U*) for each individual, and the probabilities *φ*_*w*_ for each weight are calculated for the given parameters.
2. For each component *k*_*w*_ of the generalized degree of each individual, a binomial process is performed with the probability *φ*_*w*_. The outcome of this process replaces *k*_*w*_ as the residual contact number.
3. For each individual, a Bernoulli trial is performed with the probability *v*(***k***;*β*, *U*). If the outcome is a success, the individual is removed from the list with all their contacts, otherwise they remain.
4. The base reproduction number is computed in the residual list and saved as the post-vaccination reproduction number *R*^*v*^. The vaccine coverage *V* is deduced from the length of the residual list. The efficiency *E*^*v*^ is calculated from *R*^*v*^ and *V*.

## Results

**Fig 1:**
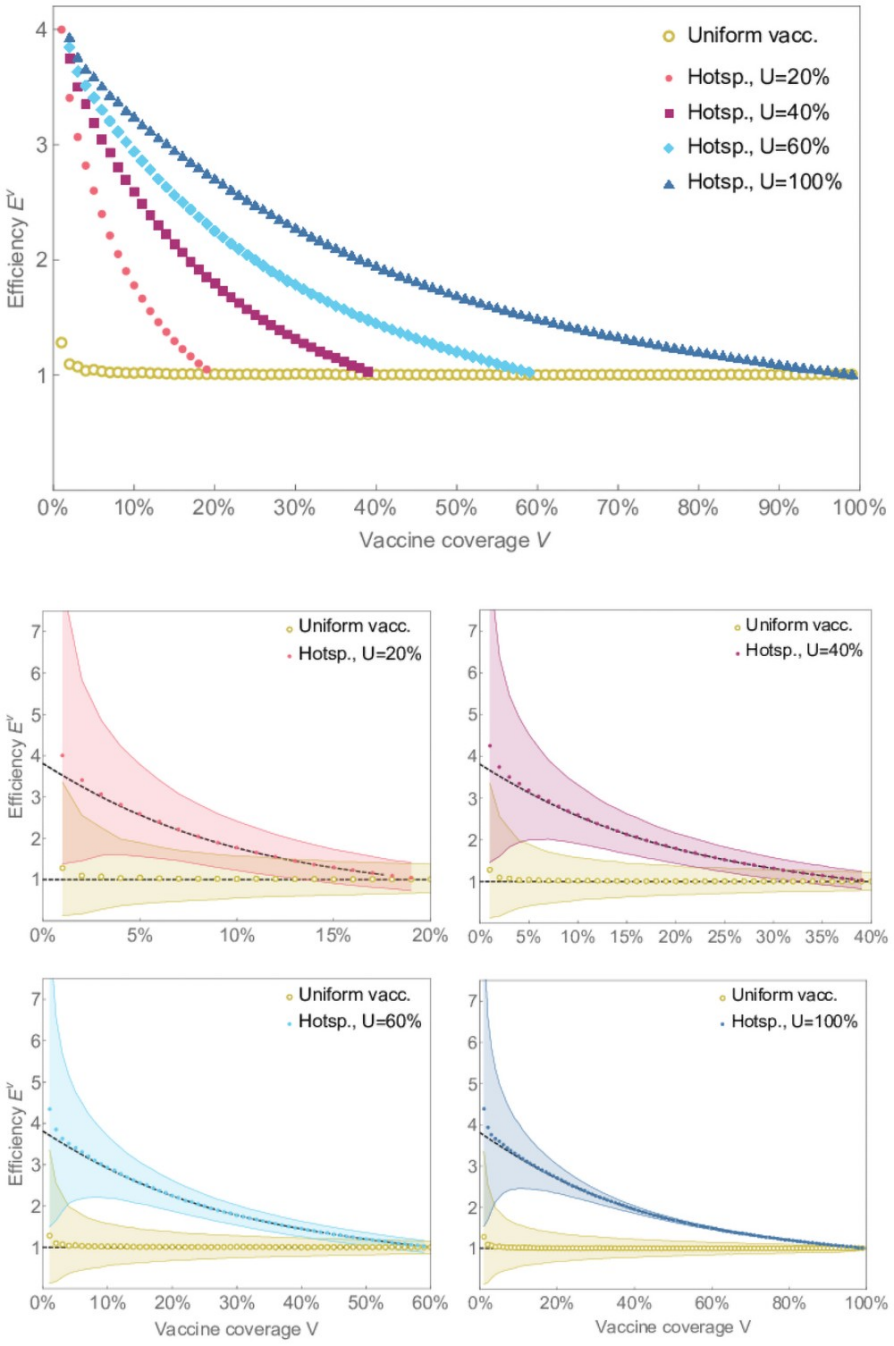
Efficiency of hot-spotting strategy. The efficiency for the hot-spotting strategy for *R*_0_ = 1.5 with various app usage rates are compared with the uniform vaccination in relation to vaccine coverage. The dots represent the mean efficiency obtained from 10,000 simulations for each dot. The shaded regions show the intervals that capture 90% of all simulation results. The dashed lines in the bottom figures show the expected efficiency obtained analytically.

Our simulation results show that the hot-spotting strategy is one to four times more efficient than the uniform vaccination strategy (Fig 1). The difference in efficiency between the two strategies is most pronounced when the vaccine coverage *V* is small, since in this case, high exposure individuals are likely to be vaccinated first under a hot-spotting strategy. As the vaccine coverage increases, hot-spotting becomes indistinguishable from the uniform strategy and its efficiency approaches 1. Indeed, when the vaccine coverage equals the app usage rate all app users are vaccinated. By our model assumptions, the app using population have the same contact patterns as the general population, and so vaccinating all app users is equivalent to uniformly vaccinating the same fraction of the population.

We also find that hot-spotting retains its lead in efficiency across a wide range of values for *U*, the rate of app usage in the population (Fig 1): vaccinating individuals with more exposure always provides more population protection for fewer vaccines expended, even if public health cannot reach all of the high-exposure individuals. The success of digital contact tracing technologies has been hampered by inadequate uptake rates [42, 43]. Our simulations show that our proposal doesn’t suffer from this issue, at least from the perspective of efficient use.

**Fig 2:**
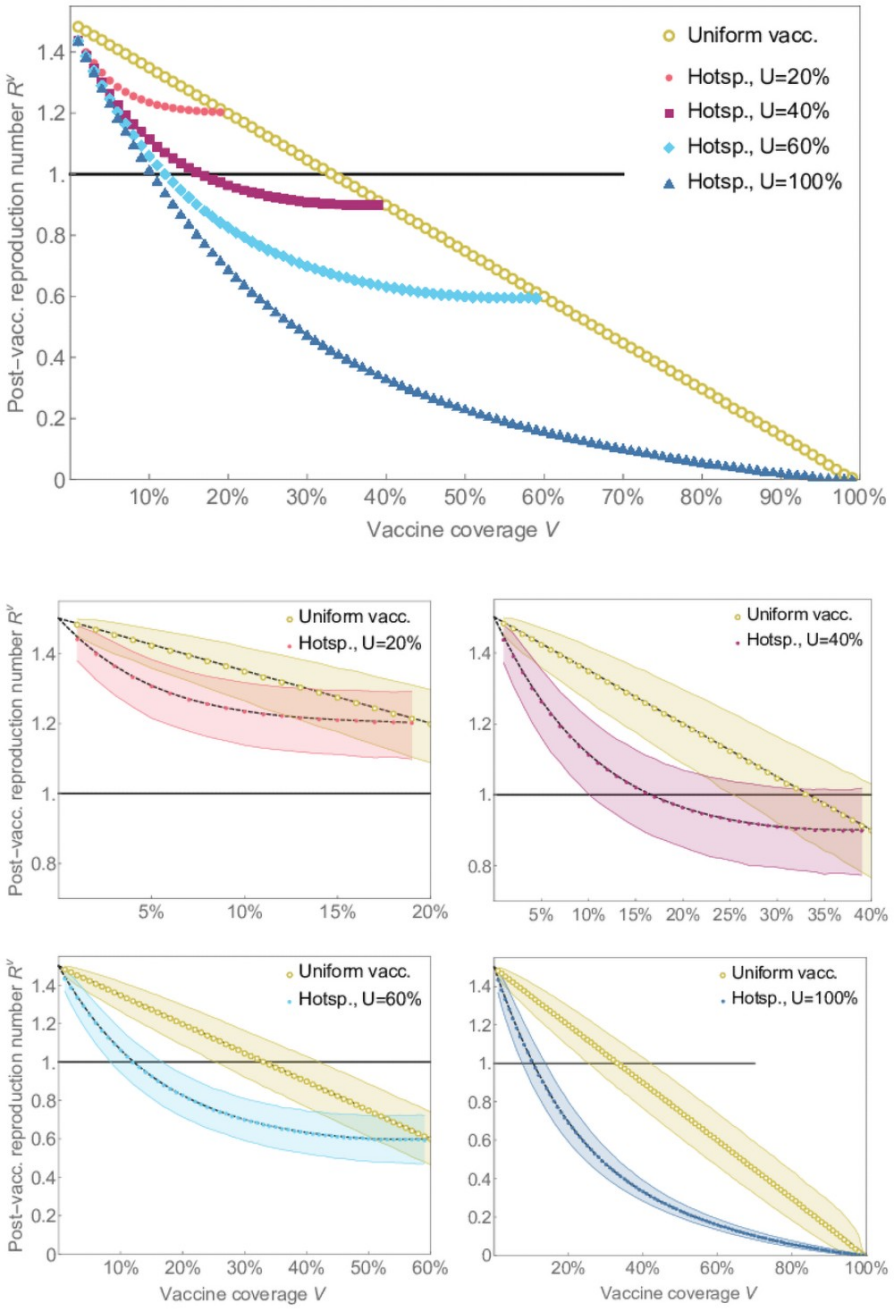
The post-vaccination reproduction numbers for the hot-spotting strategy. The post-vaccination reproduction numbers for the hot-spotting strategy for *R*_0_ = 1.5 with various app usage rates are compared with the uniform vaccination in relation to vaccine coverage. The dots represent the mean reproduction number obtained from 10,000 simulations for each dot. The shaded regions show the intervals that capture 90% of all simulation results. The dashed lines in the bottom figures show the expected post-vaccination reproduction numbers obtained analytically.

**Fig 3:**
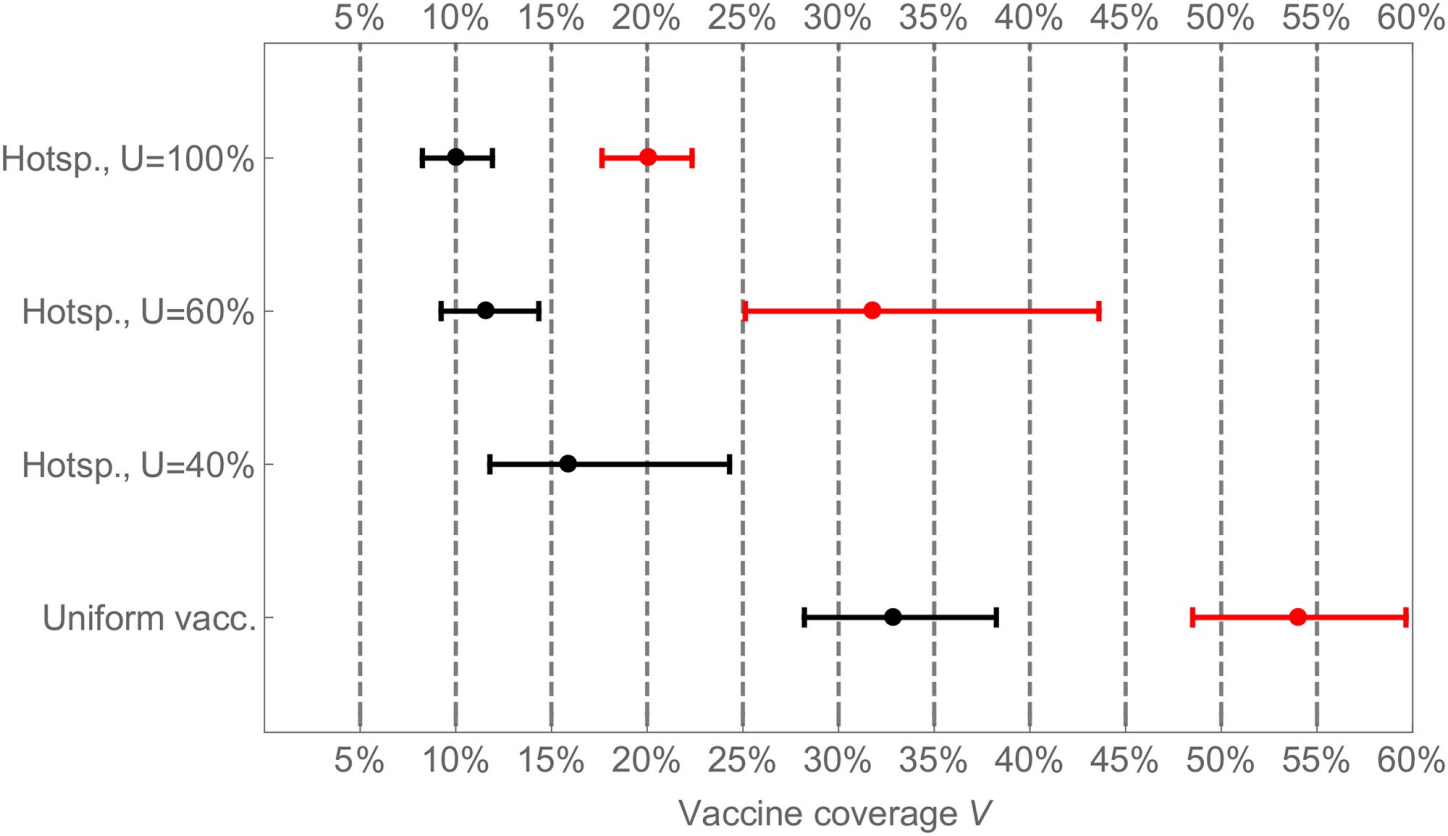
Necessary vaccine coverage to achieve herd immunity. The necessary vaccine coverage to bring the reproduction number from its initial value *R*_0_ = 1.5 (black) or *R*_0_ = 2.2 (red) to the herd immunity threshold *R*^*v*^ = 1 is demonstrated for the uniform and hot-spotting strategy for various app usage rates. The dots represent the mean value from our simulations, whereas the intervals show the range that captures 1*σ* (68.27%) of all simulation results. If *R*_0_ = 2.2 and *U* = 40%, vaccinating only the app users would not be enough to reach herd immunity.

These trends are also reflected in the impact of the two strategies on the post-vaccination reproduction number (Fig 2). Under the uniform strategy, the post-vaccine reproduction number declines in direct proportion to the vaccine coverage. But for the hot-spotting strategy, the post-vaccine reproduction number declines very rapidly for very low vaccine coverage before eventually converging to the same level as the uniform strategy, when enough individuals are vaccinated. The greatest relative impact is achieved by vaccinating 20-40% of the app users. Finally, when app usage rates are high enough, hot-spotting achieves herd immunity with fewer than half as many doses. In Fig 3 we show that the significantly lower herd immunity thresholds persist at a higher *R*_0_ value of 2.2, which is in the middle range of estimates for the 2009 influenza A/H1N1 pandemic in the USA [41].

In general, the necessary vaccine coverage *V*_*H*_ required to reach herd immunity is given by the solution of the equation

VH=1EXv(VH)(1-1R0).

For a vaccine allocation strategy with *E*^*v*^ > 1, this equation shows that *V*_*H*_ can be reduced through such strategy by a factor of EXv(VH).

## App-using subnetwork

In our model we assumed that app users are uniformly distributed in the network and that there is no preferential attachment. The actual contact patterns of app users need not satisfy this condition in a given population and can have meaningful impacts on the efficiency of the hot-spotting strategy. For example, if very few high exposure individuals are app users the hot-spotting strategy might be less efficient than the uniform strategy. Or if there is very little mixing between the app using and non-app using populations.

In order to model non-uniformly distributed app-using populations one can make use of mixing matrices (cf. [44]). More specifically, one needs the following information about the network: The distributions of generalized degree for both app users, $\left\{q_{k}^{A}\right\}$, and non-users $\left\{q_{k}^{N}\right\}$; and, for each weight *w*, a mixing matrix $e_{ij}^{w}$ where each entry is the fraction of edges in the network having weight *w* and joining a vertex of type *i* to one of type *j*, *i*, *j* ∈ {*A*, *N*}. The assumptions made in our model are that the app using and non-app using population have the same generalized degree distribution, $q_{k}^{A}=q_{k}^{N}$, and that the mixing matrices for an app usage rate of *U* are $e_{AA}^{w}\propto U^{2}$, $e_{AN}^{w}\propto U\left(1-U\right)$ and $e_{NN}^{w}\propto {\left(1-U\right)}^{2}$.

One can readily extend our formula obtained above for the expected post-vaccination reproduction number $E[R^{v}]$ to a non-uniformly distributed app-using subnetworks along the same lines as [44]. The more difficult part is finding data to determine the network variables for the model. For exposure notification apps operating under the Google/Apple framework there is currently no centralized collection of contact data and so no direct way for public health authorities to measure these variables. In principle such a functionality could be included in the future, though concerns over privacy must be addressed.

The Digital Global Health & Humanitarianism Lab carried out case studies on uptake of digital contact tracing apps in 5 countries: Iceland, Cyprus, Ireland, Scotland, and South Africa [45]. Their research indicated a number of individual and system-level factors influencing uptake, such as concerns around data collection, sense of community, accessibility and trust in institutions. While these results likely indicate a degree of preferential attachment, it is otherwise impossible to extract quantitative network statistics. This is especially true since the same social factors could lead to very different network structures in different communities. In particular, there is no a priori reason to assume that app users have more or less exposure on average than non-users.

In the absence of data to inform the structure of the app-using population we limited the scope of this work to the uniform case. We can, however, briefly comment on some features of the general case.

In the limit when *all* app users are vaccinated the residual network of unvaccinated individuals is exactly the subnetwork of non-users. The post-vaccination reproduction number therefore agrees with the basic reproduction number, $R_{0}^{N}$, of the non-user subnetwork. If there is a high degree of preferential attachment between non-users and/or they have much higher overall exposure it could be that $R_{0}^{N}>R_{0}$, leading to an expected efficiency of less than 1 for the hot-spotting strategy. In other words, in this scenario it would have been better to vaccinate the same number of people using the uniform strategy. This doesn’t necessarily imply that the uniform strategy is always better than hot-spotting in this population. The hot-spotting strategy initially prioritizes the highest exposure app-users and so can still achieve a higher efficiency than the uniform strategy when vaccinating a smaller fraction of the app-using population, assuming there are high exposure app-users. Conversely, in cases where $R_{0}^{N}<R_{0}$ the efficiency of the hot-spotting strategy is always more efficient than the uniform strategy.

## Concluding remarks

An important limitation of our modelling is the underlying data used to construct the model network [9], which does not take into account any changes in contact patterns as a result of disease spread. Moreover, in both our theoretical work and our simulations we have assumed that there is no distinction between a potentially infectious contact and the contacts detected by Bluetooth exposure notification apps. Our percolation model is not equipped to analyze the time evolution of concurrent vaccination and disease spread. Instead, it is limited to describing the structural properties of the network of unvaccinated individuals. We have also limited our modelling to the hot-spotting strategy deployed in isolation, rather than as one part of a broader vaccine allocation strategy. Finally, we have only considered the impact of vaccination strategies on overall disease spread. Other factors such as mortality or health care system strain are, of course, also important features to consider when comparing vaccination strategies.

These simplifying assumptions could be relaxed in future work with more detailed agent-based simulations that test the generality of the new theory we have introduced. However, we note that a diverse collection of previous literature already finds that prioritizing individuals based on their contact patterns can be highly effective [8, 13–15, 46] at reducing the burden of infectious disease. We have built upon this literature by (1) proposing a measure *E*^*v*^ of the relative efficiency of different strategies, (2) showing that existing COVID-19 digital contact tracing technology allows the measurement of epidemiologically important quantities without violating privacy, (3) proposing a new vaccination strategy based on these measures that is technically feasible to implement, and (4) introducing novel analytical techniques and simulations that (5) demonstrate how hot-spotting is dramatically more efficient than uniform allocation.
